# Cost-effectiveness analysis of pembrolizumab in combination with chemotherapy compared with chemotherapy alone as first-line treatment for patients with advanced biliary tract cancer in China

**DOI:** 10.1186/s12885-023-11255-w

**Published:** 2023-09-04

**Authors:** Zhiwei Zheng, Ling Fang, Hongfu Cai

**Affiliations:** 1 Department of Pharmacy, Cancer Hospital of Shantou University Medical College, Shantou, 515041 China; 2 Department of Pharmacy, Fujian Medical University Union Hospital, Fuzhou, 350001 China

**Keywords:** Pembrolizumab, Cost-effectiveness analysis, Biliary tract cancers, Chemotherapy, Partitioned survival model

## Abstract

**Objective:**

The objective of this study is to evaluate the cost-effectiveness of adding pembrolizumab to the standard first-line therapy of advanced biliary tract cancer (BTC) with gemcitabine and cisplatin from the perspective of the Chinese healthcare system.

**Methods:**

The partitioned survival model developed from clinical data obtained in The KEYNOTE-966 trial served as the basis for a simulation in the TreeAge Pro 2011 software. The objective of the research was to estimate the 10-year life expectancy and total healthcare costs of patients with BTC, utilizing primary outcomes that evaluated costs, quality-adjusted life years (QALYs), and incremental cost-effectiveness ratio (ICER). To establish the willingness-to-pay (WTP) threshold, the 2022 Chinese per capita gross domestic product (GDP) of $37304.346/QALY was adopted. Furthermore, sensitivity analysis was conducted to ascertain the study’s results under varying levels of uncertainty.

**Results:**

Compared to chemotherapy alone, the addition of pembrolizumab to chemotherapy has been shown to yield an incremental gain of 0.184 quality-adjusted life years (QALY) at an additional cost of $103940.706. This translates into an incremental cost-effectiveness ratio (ICER) of $564895.141/QALY, which exceeds the willingness-to-pay (WTP) threshold in China. One-way sensitivity analyses performed on the model recognize the utility of PD, subsequent cost, and the cost of Pembrolizumab 100 mg had a major influence on the outcomes. However, no parameter elicited an ICER lower than the willingness-to-pay (WTP) threshold.

**Conclusions:**

Based on the perspective of the Chinese healthcare system, the utilization of pembrolizumab in combination with chemotherapy as an first-line treatment option for BTC does not appear to be a cost-effective approach compared to chemotherapy as a standalone therapy.

**Supplementary Information:**

The online version contains supplementary material available at 10.1186/s12885-023-11255-w.

## Introduction

Biliary tract cancer (BTC) are a highly aggressive tumors that include cholangiocarcinomas and gallbladder cancers [1]. Although relatively rare, BTC exhibit high levels of malignancy [2]. Incidence rates of BTC are generally low (ranging from 0.35 to 2 cases per 100,000 individuals per year) in most developed countries [3]. However, in East Asian developing nations, particularly China, the incidence of biliary malignancies is significantly up to 40-times higher, representing a pressing health concern worthy of attention [4]. Currently, the etiology of cholangiocarcinoma remains uncertain, as it may be influenced by various liver and gallbladder diseases while also being potentially linked to lifestyle factors such as smoking, alcohol consumption, and obesity. In recent years, the incidence of this malignancy has shown an upward trend, possibly attributed to the improved socio-economic conditions and alterations in unfavorable dietary patterns.The majority of patients with BTC are diagnosed at an advanced stage, resulting in limited eligibility for curative-intent resection [5]. Palliative chemotherapy remains the standard treatment approach included first-line and second-line chemotherapies involving Cisplatin + Gicitabine and FOLFOX(oxaliplatin + calciumfolinate + fluorouracil), respectively [6]. However, recent years have witnessed significant developments in the treatment of BTC with the emergence of novel therapeutic targets including targeted therapy and immunotherapy [7]. Nonetheless, effective medical therapy remains a considerable challenge in the management of these hepatobiliary malignancies. Immunotherapy, such as immune checkpoint inhibitors (ICIs), has shown antitumor responses in a select group of patients [8]. Notably, current studies indicate that immunotherapy in the form of adoptive cell therapy represents a promising approach in solid tumor malignancies. In the first-line treatment of advanced BTC, combining Cisplatin + Gicitabine with durvalumab, a monoclonal antibody targeting the immune checkpoint programmed cell death ligand-1(PDL-1), has shown overall survival benefit in the phase 2 TOPAZ-1 trial [9]. Pembrolizumab, a novel programmed cell death protein 1 (PD-1) inhibitor, has demonstrated remarkable clinical efficacy in treating various solid tumors [10, 11]. Recently, in an open-label, randomized, phase 3 clinical trial (KEYNOTE-966), pembrolizumab combined with chemotherapy showed a promising improvement in the survival of patients with advanced BTC [12]. The study revealed that the median overall survival of patients treated with pembrolizumab was 12.7 months, which was significantly higher than that observed in the placebo group (10.9 months). These findings further emphasize the potential benefits of immunotherapy combined with chemotherapy and provide a foundation to advance cancer treatment strategies in the clinical setting. However, the cost-effectiveness of combining pembrolizumab with gemcitabine and cisplatin for the treatment of advanced BTC in the Chinese healthcare system is currently unknown. It is important to note that innovative medications like pembrolizumab can pose a significant economic burden on the public healthcare system. Hence, evaluating the cost-effectiveness ratio of these therapies is crucial in the formulation of therapeutic and healthcare policies, wherein economic advantages must be weighed against therapeutic effectiveness to achieve optimal outcomes with the minimum financial burden. Considering the potential benefits of pembrolizumab in combination with chemotherapy for advanced BTC, it is imperative to assess the cost-effectiveness of this treatment option. Thus, the aim of this study is to evaluate the cost-effectiveness of pembrolizumab in combination with chemotherapy as a first-line treatment option for advanced BTC from the perspective of the Chinese healthcare system.

## Methods

### Population and treatment

The present study focused on patients who are consistent with those who participated in the phase III KEYNOTE-966 clinical trial. Local patients were not included in the study. From October 4, 2019, to June 8, 2021, a total of 1564 patients were screened for eligibility, and 1069 patients were randomly assigned to two groups: the pembrolizumab group (*n* = 533) receiving pembrolizumab in combination with gemcitabine and cisplatin, and the placebo group (*n* = 536) receiving placebo with gemcitabine and cisplatin. The study protocol involved the administration of either intravenous pembrolizumab 200 mg or placebo once every three weeks. Intravenous administration of gemcitabine 1000 mg/m² and cisplatin 25 mg/m² occurred on days one and eight of three-week cycles.

The clinical trial, KEYNOTE-966, revealed that the median treatment duration for pembrolizumab and placebo groups were 6.37 months (2.79–10.84) and 5.54 months (2.53–9.69), respectively. The median number of administered cycles for pembrolizumab and placebo groups were 9 (4–16) and 8 (4–14), correspondingly. It must be further noted that 47% (253 out of 533) of the participants in the pembrolizumab group and 49% (261 out of 536) of the participants in the placebo group received subsequent anticancer therapies following treatment progression. When the disease progressed, it is recommended to administer combination therapy as a standard second-line treatment for the patient. This involves chemotherapy using the FOLFOX regimen, immunotherapy with duvacizumab, and an anti-angiogenesis inhibitor such as regorafenib. Such recommendations have been derived from consultations with clinical experts, as well as relevant clinical guidelines [13]. In our model, adverse events (AEs) of grade 3–4 were considered if they they had an incidence rate exceeding 10% in both the pembrolizumab and chemotherapy treatment arms.

### Partition survival model structure

A partition survival model(PSM) was developed to estimate the costs and clinical outcomes for patients with BTC who receive pembrolizumab plus gemcitabine and cisplatin or gemcitabine and cisplatin alone. The model considered the direct medical costs, including drug costs, adverse event management costs, and disease management costs. The clinical outcomes were expressed as total cost, quality-adjusted life years (QALYs), and the incremental cost-effectiveness ratio (ICER) gained and were based on the progression-free survival and overall survival data from the clinical trials of pembrolizumab in advanced BTC.

**Fig. 1:**
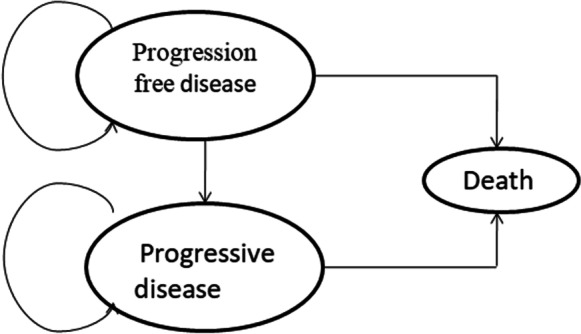
The partition survival model structure

BCTs are usually diagnosed at an advanced stage, resulting in poor prognosis,and a median OS of less than 1 year, with a 5 to 15% five-year survival rate [14]. PSM determine the number or proportion of individuals in each state by means of survival curves.PSM can directly use OS curves to estimate the number or proportion of individuals alive at a particular time point/time period and can be extrapolated statistically to predict survival data beyond the original study time frame, and OS curves can be further decomposed or partitioned into different states. In a partitioned survival model, each state has a corresponding survival curve that describes the time at which individuals move from the start of the model (initial state) to other states. Two survival curves are needed to estimate the state membership of the model when using partitioned survival models: the PFS curve and the OS curve, which are endpoint events frequently used in current cancer clinical trials.The PFS curve reflects survival data without progression, whereas the OS curve reflects overall survival data, and thus the state membership of progression can be obtained by subtracting the survival data from the OS curve and the PFS curve. The model is generally partitioned into 3 health states: Progressed free disease (PFD), Progressed Disease (PD), and Death (Fig. 1). The simulation cycle was set to three weeks in line with the KEYNOTE-966 clinical trial, with a horizon time of 10 years. It is noteworthy that in our study, a willingness-to-pay (WTP) threshold of $37304.346/QALY was utilized, which is equivalent to three times the national gross domestic product (GDP) for the year 2022 [15]. Our model was developed and conducted using the software of TreeAge Pro 2011 (Williamstown, MA, USA).

### Transition probabilities

The survival data of each treatment group were obtained by extracting information from the survival curves presented in the KEYNOTE-966 clinical trial through the use of the GetData Graph Digitizer (version 2.25).The R software was utilized to reconstruct the extracted curve, and the process involved selecting the most appropriate distribution from a range of options, including the weibull, log-logistic, log-normal, exponential, gompertz, and gamma distributions to accurately capture individual patient data [16]. Through visual inspection and rigorous statistical analysis, we determined that the weibull distribution exhibited the lowest akaike information criterion (AIC) and bayesian information criterion (BIC) values, thus making this distribution the optimal choice for predicting the long-term survival status of patients [17] (Supplementary Tables 1, Supplementary Fig. 1).

When the model is run for the trial follow-up period, survival rates are obtained directly from the survival curves, and the distribution of the number of people in each health state is obtained directly from the survival curves. When the model runs beyond the follow-up period, the survival function S(t) is calculated by using the best fit Weibull distribution to simulation the survival time. The survival function was determined through the calculation of the time transition probability, providing the expression S(t) = exp(-λt^γ^) [18]. The shape parameter (γ) and scale parameter (λ) were estimated using R software, and their respective values are presented in Table 1.

**Table 1:** Model parameters input and the range of the sensitivity analysis

Parameters	Baseline value	Range		Distribution	Source
		Minimum	Maximum		
Weibull OS survival model					
Pembrolizumab group	shape (γ) = 1.768; scale (λ) = 0.0112	-	-	-	[12]
Chemotherapy group	shape (γ) = 1.779; scale (λ) = 0.0141	-	-	-	[12]
Weibull PFS survival model					
Pembrolizumab group	shape (γ) = 1.576; scale (λ) = 0.0575	-	-	-	[12]
Chemotherapy group	shape (γ) = 1.726; scale (λ) = 0.0546	-	-	-	[12]
Drug costs ($)					
Pembrolizumab (100 mg)	2597.83	2078.26	3117.39	Gamma	[19]
Cisplatin (20 mg)	2.48	1.98	2.98	Gamma	[19]
Gemcitabine (1 g)	65.94	52.75	79.13	Gamma	[19]
Treatment-emergent adverse event (Pembrolizumab group)					
Neutropenia	0.49	-	-	Gamma	[12]
Anemia	0.28	-	-	Gamma	[12]
Thrombocytopenia	0.18	-	-	Gamma	[12]
Treatment-emergent adverse event (Chemotherapy group)					
Neutropenia	0.47	-	-	Gamma	[12]
Anemia	0.29	-	-	Gamma	[12]
Thrombocytopenia	0.20	-	-	Gamma	[12]
Cost of treatment-emergent adverse event per cycle					
Neutropenia	354	283	425	Gamma	[20]
Anemia	531	425	638	Gamma	[20]
Thrombocytopenia	1,814	1,451	2,177	Gamma	[20]
Utility					
Progression-free disease	0.76	0.61	0.91	Beta	[20]
Progressive disease	0.68	0.54	0.82	Beta	[20]
Neutropenia	0.09	0.072	0.108	Beta	[20]
Anemia	0.125	0.100	0.150	Beta	[20]
Thrombocytopenia	0.200	0.160	0.240	Beta	[20]
Other parameters					
Subsequent therapy cost per cycle	4517.85	3614.28	5421.42	Gamma	[21]
Follow-up cost per cycle	55.60	44.48	66.72	Gamma	[21]
Best supportive care	337.50	270.00	405.00	Gamma	[21]
Body surface area (m^2^)	1.72	1.38	2.06	Gamma	[22]
Discount rate	0.05	0	0.06	Gamma	[23]

### Cost and utility

The current study solely focused on the evaluation of direct medical care costs associated with cancer treatment, which included expenses related to medication, the management of severe adverse events (grade 3 and 4), follow-up costs, subsequent therapeutic expenses, and costs for best supportive care. To calculate drug costs, we utilized the Chinese health industry data platform (https://data.yaozh.com/) and established the national median price as the benchmark. Other costs were obtained from relevant literature. All costs were converted to United States dollars using the official annual average exchange rate of RMB 6.8825 to $1 as of 2022 [24]. The chemotherapy dosages prescribed were determined based on a standardized model that took into account a body weight of 65 kg and a body surface area of 1.72 m² [22].

The assessment of health-related quality of life for each health condition was conducted by utilizing utility values, which range from 0 to 1 and signify the worst and best health statuses, respectively. Due to the absence of health-related data in the KEYNOTE-966 clinical trial, the utility values employed in this model were drawn from published literature. Furthermore, the disutility of any adverse events was assessed in the model. A comprehensive display of the costs and utility values is presented in Table 1.

### Sensitivity analysis

A comprehensive analysis was conducted to explore the factors that may influence the incremental cost-effectiveness ratio (ICER) of the intervention. In order to test the robustness of the model, this study conducted a deterministic sensitivity analysis (DSA). One-way sensitivity analysis was performed by independently adjusting each input parameter by ± 20%, while varying the discount rate from 0 to 8%. The resultant tornado diagram highlights the impact of each parameter on the ICER.

Furthermore, to evaluate the robustness of our findings, a probabilistic sensitivity analysis (PSA) was conducted by executing 1000 Monte Carlo simulations. We utilized the Gamma distribution to model the cost factors and the Beta distribution to capture the utility value factors. The results of the PSA are represented through cost-effectiveness acceptability curves.

## Results

### Base-case results

In comparison to chemotherapy alone, the implementation of pembrolizumab in conjunction with chemotherapy has demonstrated an increase of 0.184 quality-adjusted life years (QALY) at an incremental cost of $103940,706.The results of the analysis indicated that the addition of pembrolizumab to gemcitabine and cisplatin in the treatment of advanced BTC was associated with an incremental cost-effectiveness ratio (ICER) of $564895.141/QALY gained. This ICER exceeded the WTP threshold of $37304.346/QALY in China, suggesting that pembrolizumab in combination with gemcitabine and cisplatin may not be a cost-effective therapy option for advanced BTC in the Chinese healthcare system. Table 2 illustrates the outcomes of the base-case analysis.

**Table 2:** The results of base-case analysis

Parameters	Cost ($)	QALYs	Incremental cost ($)	Incremental QALY	ICER ($/QALY)
Pembrolizumab group	113359.694	0.878	103940.706	0.184	564895.141
Chemotherapy group	9418.988	0.694	NA	NA	NA

*ICER* Incremental cost–effectiveness ratio, *QALY* Quality-adjusted life year, *NA* Not applicable

### Sensitivity analysis

**Figs. 2:**
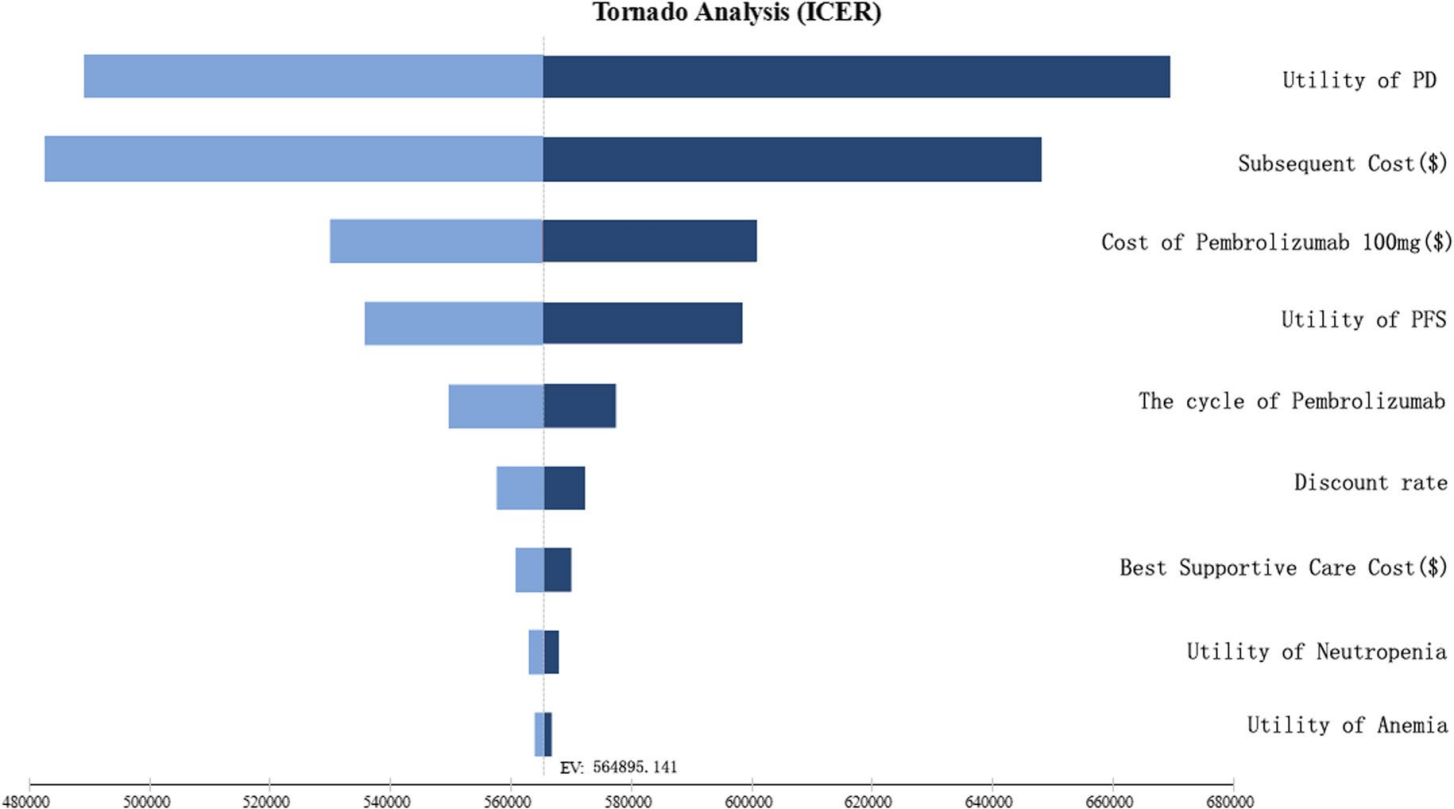
Tornado diagram of analyses

The results of the one-way sensitivity DSA analysis have been portrayed via the tornado diagram (Fig. 2). The parameters that had a major influence on the outcomes of the analysis have been identified as the utility of PD, subsequent cost, and the cost of pembrolizumab 100 mg. While the remaining parameters had a negligible impact on the results. Furthermore, it was observed that these parameters could be varied within a certain range without causing a reversal of the basic analysis results, indicating the robustness of the model. Stated differently, it can be inferred that no parameter led to an ICER that was lower than the WTP threshold.

**Figs. 3:**
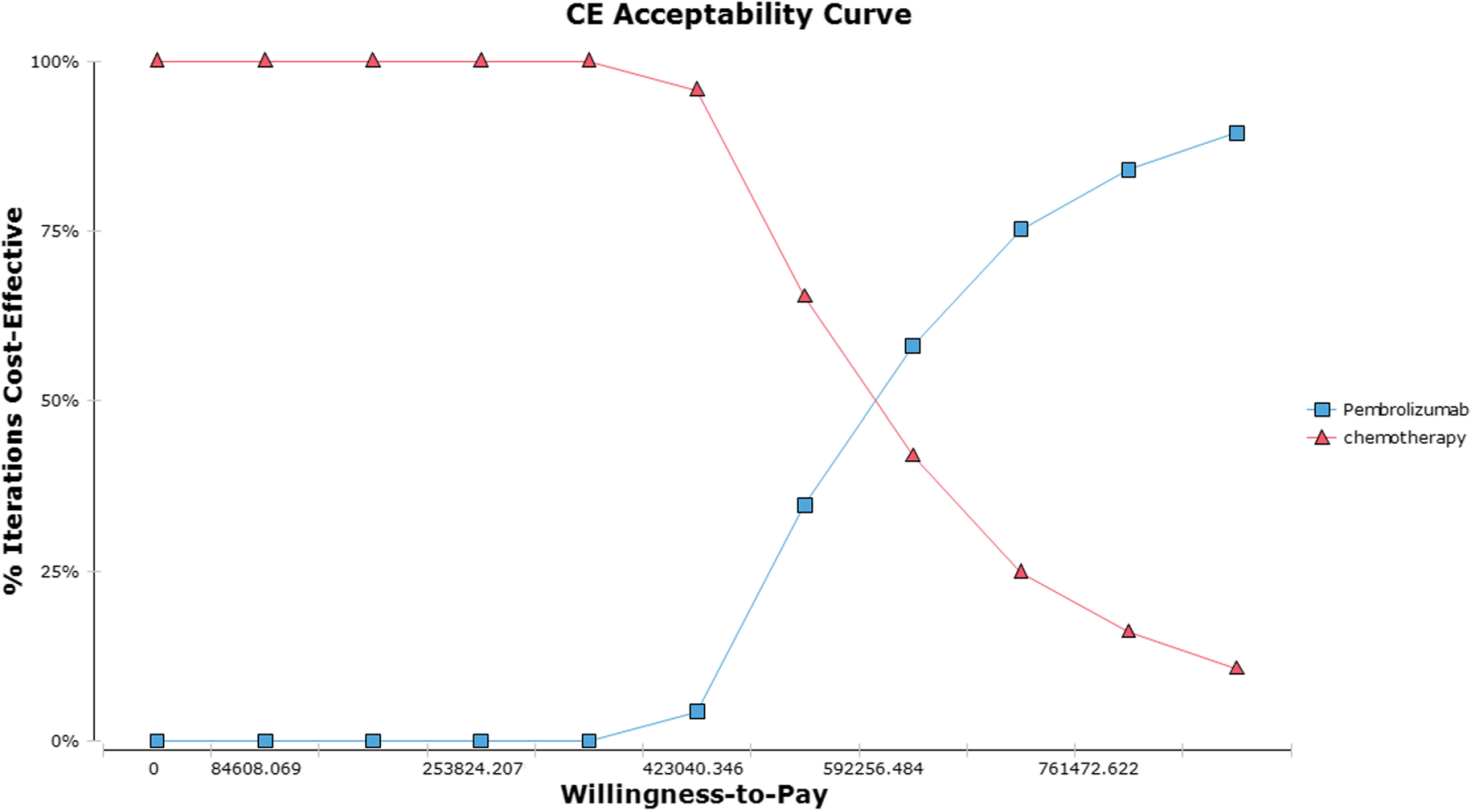
The acceptability curve of probabilistic sensitivity analysis

The acceptability curve for the cost-effectiveness of PSA is presented in Fig. 3. Notably, the cost-effective probability of pembrolizumab combined with chemotherapy in comparison to chemotherapy alone was 0% with a WTP threshold of $37304.346/QALY. However, the probability of cost-effectiveness for pembrolizumab with chemotherapy increased to 34.70% and 58.10% respectively, when the WTP threshold was raised to $507648.415/QALY and $592256.484/QALY.

## Discussion

Biliary tract cancers (BTC) constitutes a complex group of malignancies that present considerable challenges for treatment and currently offer limited therapeutic options. However, over the past years, we have observed a new epoch in the medical management of BTC with significant advancements in clinical research [25]. Our enhanced comprehension of the molecular biology of these diverse tumors has been instrumental in this progress. Despite their heterogeneity, ongoing research has led to noteworthy breakthroughs in the identification of potential treatment targets for these rare malignancies. Immunotherapy has emerged as a promising avenue for the treatment of BTC [26]. There are some clinical trials are exploring the potential of immune checkpoint inhibitors (ICIs) as a novel and effective therapeutic approach. These trials are expected to provide valuable insights and establish the safety and efficacy of ICIs for the treatment of BTC, paving the way for the development of new treatment options for these challenging cancers [27]. The findings of the KEYNOTE-966 trial contribute valuable evidence to the efficacy and safety of incorporating ICIs targeting PD-1 and PD-L1 into standard-of-care chemotherapy for the treatment of BTC. These results demonstrate a statistically significant and clinically meaningful improvement in overall survival, without any new safety concerns identified, bolstering support for the use of the pembrolizumab plus gemcitabine and cisplatin combination as a promising first-line treatment option for individuals with metastatic BTC.The adoption of pembrolizumab in cancer treatment has shown remarkable clinical benefits, particularly in advanced and metastatic tumors. However, the high cost of the drug may hinder its widespread usage, especially for patients with limited economic means. Cancer patients in China, in particular, often experience unequal access to outpatient and inpatient care due to socioeconomic disparities, which necessitates a focus on value-based oncological care [28]. To address these challenges, the assessment of economic evaluations of drugs becomes imperative, as it provides a robust and systematic approach to examine the costs and benefits associated with different treatment options. With such an approach, healthcare providers and policymakers can make informed decisions on the allocation of resources and prioritize the most effective treatments for their patients. Therefore, The primary objective of this study is to assess the cost-effectiveness of utilizing pembrolizumab, in combination with chemotherapy, as a first-line treatment approach for advanced BTC by considering the Chinese healthcare system’s perspective.

Previous research has investigated the efficacy of immune checkpoint inhibitors (ICIs) plus chemotherapy as a primary treatment for advanced BTC. The TOPAZ-1 phase 3 clinical trial revealed a significant enhancement in the overall survival of patients treated with durvalumab, in comparison to those who received chemotherapy alone [9]. However, there were some cost-effectiveness analysis indicated that implementing durvalumab as a first-line therapy for BTC patients in China may not be a cost-effective treatment [20, 21].

According to our research, pembrolizumab plus gemcitabine and cisplatin yielded an ICER of $564895.141 per QALY gained. This exceeds the commonly accepted WTP threshold of $37304.346/QALY, indicating that the use of pembrolizumab plus gemcitabine and cisplatin may not be a cost-effective treatment option within the perspective of the Chinese healthcare system. These results have important implications for healthcare resource allocation and decision-making in managing advanced BTC in China.

The sensitivity analysis results underscore the crucial role of PD utility, cost, and the cost of pembrolizumab in influencing the outcomes of chemotherapy for BTC. It is noteworthy, however, that varying any parameter within a given interval did not result in an ICER lower than the WTP threshold. Thus, the current prices of pembrolizumab are prohibitively high and further reductions are essential for the cost-effectiveness of this treatment regimen.

In our study, the primary determinant of the ICER result was found to be the utility of PD. The utility value represents the health-related quality of life associated with each particular health condition. A lower utility value implies a greater impact of ICER on the outcome, whereas a higher utility value implies a lower ICER value on the outcome. However, after adjusting the utility values of PD by ± 20%, we observed that the minimal ICER did not reach the threshold of WTP. This finding substantiates the robustness of our study outcomes.The utilization of pembrolizumab in combination with chemotherapy as an first-line treatment option for BTC does not appear to be a cost-effective approach compared to chemotherapy as a standalone therapy.

When the disease progressed, it is recommended to administer combination therapy as a standard second-line treatment for the patient. This involves chemotherapy using the FOLFOX regimen, immunotherapy with duvacizumab, and an anti-angiogenesis inhibitor such as regorafenib. Additionally, irinotecan plus capecitabine may also be utilized as subsequent anticancer therapy. Such recommendations have been derived from consultations with clinical experts, as well as relevant clinical guidelines.

We have analyses to assess the impact of different subsequent therapy regimens on treatment outcomes. Unfortunately, however, even treatment with the lowest-cost FOLFOX regimen does not yield an ICER below the WTP threshold. The choice of a specific subsequent therapy regimen therefore does not significantly affect the results of our study, as evidenced by the results of our sensitivity analyses.

We assert that cost-effectiveness analysis can serve as an effective approach in determining scientifically-sound drug pricing for cancer treatment. The exorbitant costs incurred by cancer drugs present an additional financial burden on both individuals and the healthcare system as a whole. To address this issue, China has implemented a national drug price negotiation system that leverages the health technology assessment method since 2017 [29]. This system has resulted in price reductions of up to 50% for some costly cancer drugs, consequently enabling access to treatment for a vast spectrum of patients. The cost-effectiveness analysis, used in conjunction with other relevant methods, can contribute significantly to the regulation of drug pricing and ultimately improve healthcare outcomes, particularly in the cancer treatment context [30].

We strongly advise against the use of cost-effectiveness analyses as a justification to limit access to pembrolizumab, a highly efficacious cancer therapeutic agent. Instead, we propose that such analyses be utilized to inform equitable pricing policies and enhance accessibility to drugs through healthcare insurance systems [31]. Our research endeavors to promote the utilization of effective medications and advocate for fair and accessible drug availability, rather than restricting their utilization based on cost-effectiveness considerations.

In the present study, it is important to acknowledge several limitations. Firstly, the absence of long-term follow-up data for the overall survival and progression-free survival of the KEYNOTE-966 clinical trial could potentially introduce uncertainty and influence the simulated outcomes. To account for this, the simulation period was set to 10 years to minimize any associated bias. Secondly, the disutility values and costs in the analysis only considered adverse events (AEs) of grades 3–4, with those of grades 1–2 being ignored due to their minimal impact on clinical outcomes. Sensitivity analyses, however, indicated that AEs had limited impact on the results. Finally, it is important to note that only direct medical care costs were evaluated, and indirect costs such as loss of productivity or caregiver expenses were not taken into account. Despite this limitation, our findings provide valuable insights into the economic burden of cancer treatment.

In conclusion, this study highlights the need for a careful consideration of the economic value of new treatment options in a resource-constrained healthcare system. While pembrolizumab has shown promising clinical results in advanced BTC, its cost-effectiveness in the Chinese healthcare context needs to be evaluated carefully to ensure optimal allocation of healthcare resources.

## Conclusion

According to the Chinese healthcare system, the combination therapy of pembrolizumab and chemotherapy as the first-line treatment for advanced BTC is deemed to be not cost-effective compared to the administration of chemotherapy alone. This conclusion is based on a thorough analysis of the available data concerning the cost and health outcomes associated with the two different treatment strategies.

### Supplementary Information

**Additional file 1: Supplementary Table 1.** Comparison of survival models.

**Additional file 2: Supplementary Figure 1. **A: Modes simulation visual overall survival curve of pembrolizumab arm; B: Modes simulation visual overall survival curve of chemotherapy arm; C: Modes simulation visual progression-free survival curve of pembrolizumab arm; D: Modes simulation visual progression-free survival curve of chemotherapy arm.

## Data Availability

The data used in the current investigation are available with reasonable request from the corresponding author.
