# Supplementary material for: Cost-effectiveness analysis of pembrolizumab in combination with chemotherapy compared with chemotherapy alone as first-line treatment for patients with advanced biliary tract cancer in China
Source: BMC Cancer. 2023 Sep 4;23:823. doi: 10.1186/s12885-023-11255-w (PMC10476407; doi:10.1186/s12885-023-11255-w)
Supplement: Supplementary file 1 — Additional file 1: Supplementary Table 1. Comparison of survival models. [file 12885_2023_11255_MOESM1_ESM.docx]

**Supplementary Table 1. Comparison of survival models**

|  | AIC | | BIC | |
| --- | --- | --- | --- | --- |
|  | Pembrolizumab group | Chemotherapy group | Pembrolizumab group | Chemotherapy |
| PFS |  |  |  |  |
| **Weibull** | **2284.838** | **2356.590** | **2293.395** | **2365.158** |
| Log-logistic | 2299.079 | 2374.812 | 2307.636 | 2383.380 |
| Log-normal | 2272.796 | 2343.794 | 2281.353 | 2352.362 |
| Gompertz | 2311.618 | 2396.741 | 2320.175 | 2405.310 |
| Exponential | 2311.099 | 2400.583 | 2315.377 | 2404.867 |
| Gamma | 2293.196 | 2365.455 | 2301.753 | 2374.024 |
| OS |  |  |  |  |
| **Weibull** | **3145.306** | **3224.337** | **3153.863** | **3232.905** |
| Log-logistic | 3164.952 | 3238.499 | 3173.509 | 3247.067 |
| Log-normal | 3159.279 | 3237.765 | 3167.836 | 3246.334 |
| Gompertz | 3189.552 | 3262.543 | 3198.109 | 3271.111 |
| Exponential | 3196.972 | 3270.913 | 3201.250 | 3275.197 |
| Gamma | 3157.318 | 3231.265 | 3165.875 | 3239.833 |

AIC: Akaike information criterion; BIC: Bayesian Information Criterion; OS: Overall survival; PFS: Progression-free survival;
