# Supplementary figures and images for: Cost-effectiveness analysis of pembrolizumab in combination with chemotherapy compared with chemotherapy alone as first-line treatment for patients with advanced biliary tract cancer in China
Source: BMC Cancer. 2023 Sep 4;23:823. doi: 10.1186/s12885-023-11255-w (PMC10476407; doi:10.1186/s12885-023-11255-w)

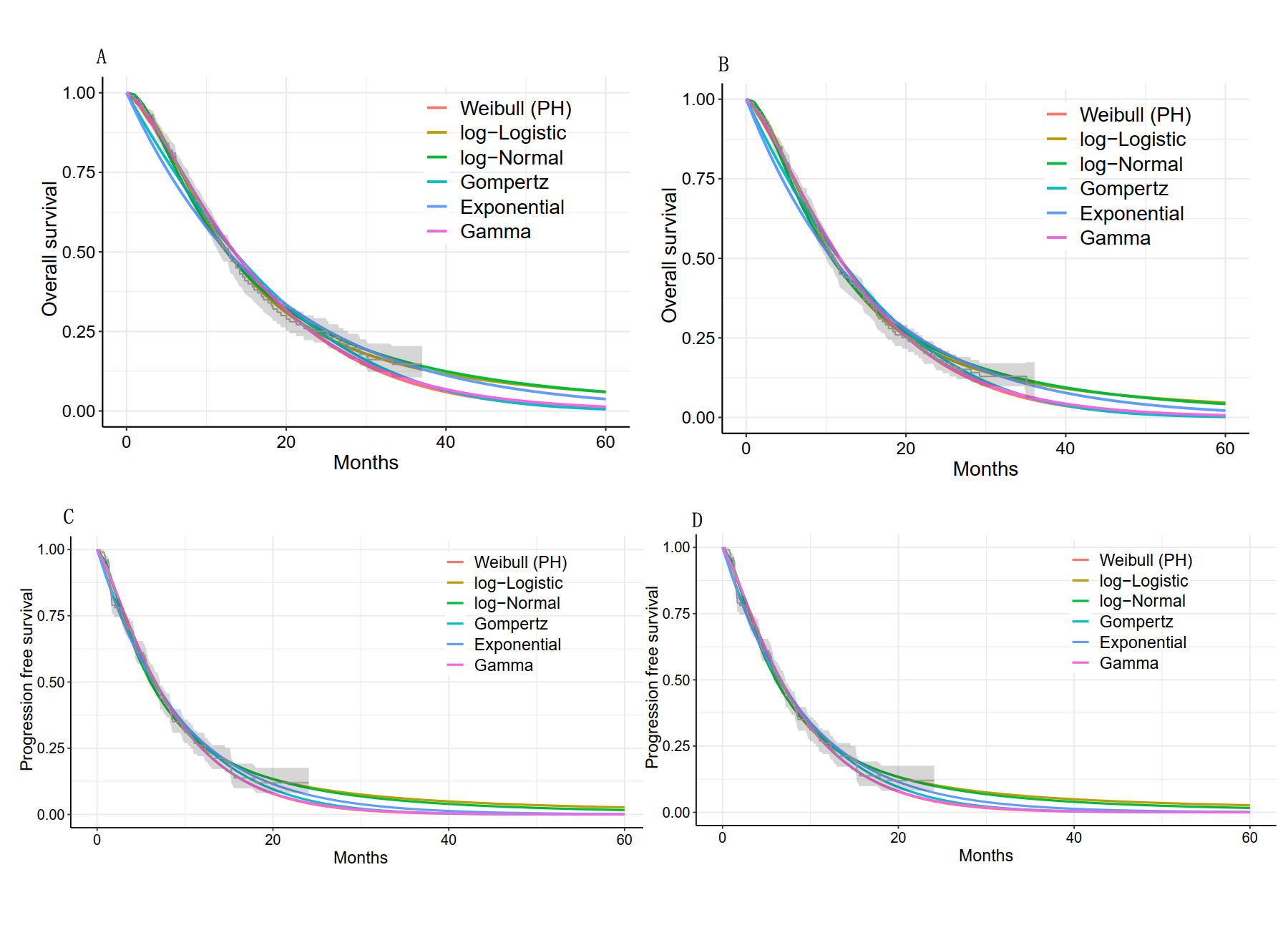

Supplement: Supplementary file 2 — Additional file 2: Supplementary Figure 1. A: Modes simulation visual overall survival curve of pembrolizumab arm; B: Modes simulation visual overall survival curve of chemotherapy arm; C: Modes simulation visual progression-free survival curve of pembrolizumab arm; D: Modes simulation visual progression-free survival curve of chemotherapy arm. [file 12885_2023_11255_MOESM2_ESM.png]
